# Effect of *P. corylifolia* on the pharmacokinetic profile of tofacitinib and the underlying mechanism

**DOI:** 10.3389/fphar.2024.1351882

**Published:** 2024-04-08

**Authors:** Yu Wang, Quan Zhou, Huihui Wang, Wei Song, Jianfeng Wang, Abdullah Al Mamun, Peiwu Geng, Yunfang Zhou, Shuanghu Wang

**Affiliations:** Key Laboratory of Joint Diagnosis and Treatment of Chronic Liver Disease and Liver Cancer of Lishui, Wenzhou Medical University Lishui Hospital, Lishui People’s Hospital, Lishui, Zhejiang, China

**Keywords:** *P. corylifolia*, drug-drug interaction, cytochrome P450, pharmacokinetics, molecular docking

## Abstract

This work aimed to explore the mechanisms underlying the interaction of the active furanocoumarins *in P. corylifolia* on tofacitinib both *in vivo* and *in vitro*. The concentration of tofacitinib and its metabolite M8 was determined using UPLC-MS/MS. The peak area ratio of M8 to tofacitinib was calculated to compare the inhibitory ability of furanocoumarin contained in the traditional Chinese medicine *P. corylifolia* in rat liver microsomes (RLMs), human liver microsomes (HLMs) and recombinant human CYP3A4 (rCYP3A4). We found that bergapten and isopsoralen exhibited more significant inhibitory activity in RLMs than other furanocoumarins. Bergapten and isopsoralen were selected to investigate tofacitinib drug interactions *in vitro* and *in vivo*. Thirty rats were randomly allocated into 5 groups (*n* = 6): control (0.5% CMC-Na), low-dose bergapten (20 mg/kg), high-dose bergapten (50 mg/kg), low-dose isopsoralen (20 mg/kg) and ketoconazole. 10 mg/kg of tofacitinib was orally intervented to each rat and the concentration level of tofacitinib in the rats were determined by UPLC-MS/MS. More imporrantly, the results showed that bergapten and isopsoralen significantly inhibited the metabolism of tofacitinib metabolism. The AUC_(0-t)_, AUC_(0-∞)_, MRT_(0-t)_, MRT_(0-∞)_ and Cmax of tofacitinib increased in varying degrees compared with the control group (all *p* < 0.05), but CLz/F decreased in varying degrees (*p* < 0.05) in the different dose bergapten group and isopsoralen group. Bergapten, isopsoralen and tofacitinib exhibit similar binding capacities with CYP3A4 by AutoDock 4.2 software, confirming that they compete for tofacitinib metabolism. *P. corylifolia* may considerably impact the metabolism of tofacitinib, which can provide essential information for the accurate therapeutic application of tofacitinib.

## 1 Introduction

Multiple drugs taken together cause DDIs, which further produce numerous side effects. In addition, DDIs may further induce adverse drug reactions (ADRs) (Zhang et al., 2020). Several drugs are frequently administered to patients with multiple disorders to boost their therapeutic effects. However, drug interactions may not always work and raise the risk of severe consequences or death. The primary factor contributing to DDIs is alterations in pharmacokinetics and pharmacodynamics (Aronson, 2004). Growing evidence indicates that the modulation of cytochrome P450 enzymes (CYP450s) through inhibition and induction can alter the pharmacokinetic characteristics of drugs, serving as a fundamental mechanism for drug interactions (Lin and Lu, 1998; Lin, 2006). Most drugs pass through metabolism by CYP oxidation (Hardman, 2001). The CYP3A4 enzyme found in the liver and intestines is highly prevalent and plays a crucial role in metabolizing approximately half of all drugs available on the market (Zhou, 2008). There is increasing evidence that drugs and/or exogenous substances stimulate or obstruct the activity and generation of CYP3A4, resulting in toxicity (Sabers, 2008). The combination of tofacitinib and ketoconazole, a CYP3A4 inhibitor, may prolong the QT interval and result in arrhythmia and mortality (Mathews et al., 1991). Accumulating evidence indicates that DDIs significantly contribute to preventable disease and death in modern healthcare (Day et al., 2017).

Tofacitinib (CP-690550) is a small-molecule oral JAK kinase inhibitor that can be used for long-term treatment (Palasik and Wang, 2021). Multiple studies have revealed that tofacitinib obstructs intracellular JAK kinase, cytokine receptor recruitment and STAT phosphorylation to hinder STAT from being activated into the nucleus, impede intracellular JAK pathway signaling, alleviate immune cell activation and pro-inflammatory factor release and inflammatory cascade (Meyer et al., 2010; Dhillon, 2017). Rheumatoid arthritis (RA) is reagred as a systemic chronic immune-mediated inflammatory disorder that affects multiple joints throughout the body. The JAK signal transduction factor and the transcription activation pathway play a crucial role in the pathogenesis of RA. Prior studies have revealed that tofacitinib can remarkably reduce bone marrow edema in patients with RA and ameliorate the progression of structural impairment (Conaghan et al., 2016). Tofacitinib has been approved for the treatment of RA in both the United States and Europe (Karaman et al., 2008).

The origins of traditional medicine for the treatment of diseases may be tracked over several thousands of years. Traditional Chinese Medicine is frequently combined with synthetic drugs to treat diseases in modern medicine. *P. corylifolia*, a traditional Chinese medicine, exhibits remarkable anti-inflammatory actions by modulating inflammatory signaling pathways, mitigating the generation and secretion of inflammatory mediators and suppressing the infiltration of inflammatory cells (Chen et al., 2023). Current evidence indicates that *P. corylifolia* potentially delivers therapeutic applications in treating inflammatory diseases including RA (Pai et al., 2021; Wang et al., 2022). A pioneering study has revealed that psoralen possesses remarkable anti-inflammatory properties and enhances the proliferation of MDSCs (Pai et al., 2021). Due to the significance of tofacitinib in treating RA and the anti-arthritis potential of psoralen, drug interaction is essential. *P. corylifolia* contains coumarins (Alam et al., 2018), which exert multiple pharmacological actions including anti-inflammatory, anti-tumor, anti-bacterial and antioxidant properties (Sharifi-Rad et al., 2021). The compounds found in *P. corylifolia* such as psoralen, isopsoralen, bergapten, psoralidin, xanthotoxol, and 8-methoxypsoralen, have been proven to possess diverse pharmacological actions including anti-inflammatory and anti-oxidation properties (Chen et al., 2023). Resarch suggests that psoralen may efficaciously alleviate ulcerative colitis inflammation (Zx, 2020). In addition, psoralen obstructs MMP-13 protein synthesis, promoting chondrocyte growth and protecting against TNF-α-induced gene dysregulation (Wang et al., 2019). More importantly, psoralen can decrease MMP-1/2/3/9/12/13 gene expressions and attenuate MMP-13 protein synthesis, which may enhance chondrocyte proliferation and cartilage-associated gene expression. In addition, psoralen protects chondrocytes against TNF-α-induced gene expression abnormalities. Studies have indicated that isopsoralen can effectively mitigate inflammation by suppressing the release of inflammatory factors *in vivo* and *in vitro* (Kong et al., 2017; Han et al., 2021; Liang et al., 2021). Bergapten (5-methoxy-psoralen) has been proven to have promising anti-inflammatory and analgesic actions (Yeo et al., 2000).

Furanocoumarins are commonly found in a variety of plants in nature. Certain furancoumarins can interact with the intestinal cytochrome P450 isomer CYP3A4, which has been determined in extensive research (Yeo et al., 2000; Nijsten and Stern, 2003; Stern, 2012). Furanocoumarins in grapefruit may strongly inhibit CYP3A4, which might affect the pharmacokinetic characteristics of multiple drugs and induce drug interactions (Ohnishi et al., 2000). Furanocoumarins in *P. corylifolia* interact with CYP3A4, which can alter pharmacokinetics and pharmacodynamics. Pioneering research revealed that resveratrol could increase plasma exposure to tofacitinib by impeding CYP3A4 enzyme (Ye et al., 2023). Tofacitinib used with moderate or severe renal and hepatic impairment must now be adjusted to 5 mg per day orally (Vyas et al., 2013). The primary adverse effects of tofacitinib included infection, gastrointestinal disturbances including perforation, increased liver enzyme levels and dyslipidemia (Moriana et al., 2022). Prolonged or high-dose administration of tofacitinib for treating RA elevates the likelihood of opportunistic infections related to immunosuppression (Beaugerie et al., 2020) and susceptibility to cardiovascular disease and malignancies (Charles-Schoeman et al., 2023; Curtis et al., 2023). Tofacitinib has been found to promote herpes zoster infection in a dose—and age-dependent manner (Beaugerie et al., 2020). A study conducted by Cox revealed that the augmented risk of severe infection in patients with RA treated with tofacitinib was attributed to the dosage of the drugs (Cohen et al., 2020). Tofacitinib dose limits may lower expectations when combined with other medicines due to tolerability and safety (Serhal and Edwards, 2019). *P. corylifolia*, a new RA medication, requires clinical and product development. In order to understand the underlying mechanism of DDIs, furocoumarins in *P. corylifolia* need to be investigated in combination with other drugs.

In this study, UPLC-MS/MS was utilized to determine tofacitinib and its metabolite M8 and to investigate the interaction of furocoumarin in psoralen in rat liver microsomes (RLMs), human liver microsomes (HLMs) and recombinant human CYP3A4 (rCYP3A4). First, eight furocoumarins in psoralen were evaluated for inhibitory activity and bergapten and isopsoralen were shown to be significant inhibitors. Berkapten and isopsoralen inhibited tofacitinib in RLMs, HLMs, and rCYP3A4, with a higher pre-inhibitory impact. Therefore, we investigated the *in vivo* interactions of bergapten, isopsoralen and tofacitinib. Our study focused on the pharmacokinetic properties of tofacitinib and the suppressory effects of bergapten and isopsoralen on tofacitinib. The binding interaction of bergapten and furanocoumarin with rCYP3A4 was finally confirmed employing molecular docking analysis to understand and anticipate the probability of interaction.

## 2 Materials and methods

### 2.1 Chemicals, reagents and biological samples

Tofacitinib (purity >98%) was purchased from Beijing InnoChem Science & Technology Co., Ltd. Psoralen, isopsoralen, psoralidin, bergapten, 8-methoxypsoralen and xanthotoxol were purchased from Chengdu Must Bio-technology Co., Ltd.Midazolam (internal standard (IS); purity >98%) was purchased from the Tianjin King York Pharmaceutical Co. Ltd. (Tianjin, China). HLMs was purchased from Wuhan PrimeTox biomedicine technology Co., Ltd. (Wuhan, China). rCYP3A4 was gifted from Beijing Hospital. Acetonitrile and methanol were bought from Merck Co., Ltd. (Darmstadt, Germany). Carboxymethylcellulose sodium salt (CMC-Na) was purchased from Sinopharm Chemical Reagent Co., Ltd. (Shanghai China). Formic acid was bought from Sigma-Aldrich (St. Louis, MO, United States). Ultrapure water was obtained using a Milli-Q water purification system (Millipore, Billerica, MA, United States). RLMs was prepared in our laboratory. All other chemicals and biological substances were of analytical grade or higher.

### 2.2 Instruments and operation conditions

The determination of tofacitinib parameters was executed by employing a UPLC-MS/MS system, which possessed an ACQUITY I Class UPLC and a XEVO TQD triple quadrupole mass spectrometer (Waters Corp., Milford, MA, United States). Chromatographic analysis of tofacitinib was conducted with a CORTECS C18 column (2.1 × 50 mm, 1.6 µm) maintained at 40°C. The mobile phase included 0.1% formic acid, 5 mM ammonium formate and acetonitrile. Acetonitrile was eluted from 10% to 30% over 0.5–1.0 min; maintained at 95% for 1.0–2.0 min and then decreased to 10% over 2.0–2.3 min. The flow rate was 0.4 mL/min and the total run time was 3 min. Multiple positive reactions were monitored in mass scan mode. The parent ion and daughter ion were m/z 313.18→149.03 for tofacitinib, m/z 299.16→98.10 for tofacitinib M8 and m/z 325.98→291.07 for IS. Tofacitinib, M8 and IS were defined as 40 V, 40 V, and 50 V cone voltages and 30 V, 30 V, and 26 V collision energies, respectively.

### 2.3 The inhibitory effect of bergapten and isopsoralen on tofacitinib *in vitro* and the mechanism of action

Tofacitinib, 30 mg/mL RLMs, 20 mg/mL HLMs, or 2.68 mg/mL rCYP3A4, 0.1 mM PBS (pH7.4), 20 mM (Reduced) Nicotinamide Adenine Dinucleotide Phosphate (NADPH), bergapten or isopsoralen, or not included were added to 200 μL incubation. 100 μM psoralen, isopsoralen, psoralidin, bergapten, 8-methoxypsoralen and xanthotoxol were incubated with 0.3 mg/mL RLMs. Bergapten and Isopsoralen were found to have high RLMs inhibitory activity after determining the content of M8, a metabolite of tofacitinib. To determine IC_50_, the concentrations of bergapten and isopsoralen used were 0.01, 0.1, 1, 5, 10, 50, and 100 μM and tofacitinib was used at concentrations approximating *Km* values. To determine the inhibitory characteristic of Ki, tofacitinib was incubated at 1/4, 1/2, 1, 2 times the *Km* value and bergapten and isopsoralen at 1/4, 1/2, 1, 2 times the IC_50_ value. All except NADPH were mixed on ice. Afterward, the mixture was incubated at 37°C for 5 min. Next, the incubation was extended for an additional 30 min following the addition of NADPH to initiate the reaction. Acetonitrile containing midazolam was added for quenching and 200 ng/mL midazolam was used as the normal solution. After vortexing, the mixture content was adequeatly centrifuged at 13,000 rpm for 5 min and 150 μL of the supernatant was stored in a sample injection bottle for UPLC-MS/MS analysis.

### 2.4 Molecular docking simulations

The X-ray crystal structure of CYP3A4 (PDB ID: 2J0D) was found using PDB (Protein Data Bank, http://www.rcsb.org/pdb). In Chem3D Ultra 14.0, the three-dimensional structure of tofacitinib, bergapten and isopsoralen were optimized for energy minimization. The smile file for each ligand was then converted to PDBQT format utilizing AutoDockTools-1.5.6 software (AutoDock 4.2 software, The Scripps Research Institute, United States). Before the docking analysis, the CYP3A4 protein structure underwent modifications including removing water, the primary inhibitor and ligand molecules. After adding hydrogen atoms and Kollman charge, AutoDockTools-1.5.6 converted the structure to PDBQT. This ligand docking selected the standard precision mode, which pairs the ligand to the active site of the target protein. For the 10-GA docking simulation, Lamarque GA with default settings was employed. Protein-ligand interactions were studied by the protein-ligand Interaction Profile and PyMOL.

### 2.5 The pharmacokinetic interactions of tofacitinib with bergapten and isopsoralen *in vivo*

Male Sprague Dawley (SD) rats (average weighing 220–250) g were purchased from the laboratory animal center of Wenzhou Medical University, Zhejiang Province, China. Animals were maintained in a controlled environment with a 12 h light-dark cycle at 20°C–25°C and 55% ± 15% relative humidity. Diet intake was not allowed for a period of 12 h prior to the experiment. Then, water was allowed to be free and food was provided after the experiment. All the experimental procedures and protocols were reviewed and approved by the animal ethics committee of Wenzhou Medical University according to the guide for the care and use of laboratory animals (xmsq2021-0409). 36 male rats aged 8–10 weeks were randomly selected and divided into six groups for gavage. The group which was orally administered 40 mg/kg ketoconazole and 10 mg/kg tofacitinib acted as the positive control group and the group which was only orally administered 10 mg/kg tofacitinib acted as the negative group. Another four groups were orally administered 20 mg/kg bergapten, 50 mg/kg of bergapten, 20 mg/kg of isopsoralen and 50 mg/kg of isopsoralen, respectively, except 10 mg/kg of tofacitinib. Blood (300 μL) was collected from the tail veins into 1.5 mL tubes at 5, 15, and 30 min and 1, 2, 3, 4, 6, 8, 12, and 24 h after administering the drugs. The supernatant was collected and stored at −20°C after centrifugation at 4000 rpm for 10 min. The samples were restored to RT before analysis. 50 μL sample was accurately drawn to 1.5 mL of EP tube, 150 μL of midazolam internal standard solution (200 ng/mL) was added to the tube, then placed and vortexed for 15 s. After centrifugation at 13, 000 rpm for 15 min, 150 µL of supernatant was prepared for the UPLC-MS/MS system to analyze.

### 2.6 Data analysis

Pharmacokinetic data were analyzed by a non-compartmental method using Drug and Statistics (DAS) software (version 3.2.8). The plasma concentration at different times was expressed as mean ± SD and the drug concentration-time curve was obtained based on the mean concentration of 6 mice assessed at each time-point. *Km*, *Vmax,* and IC_50_ values were performed using Prism software v.8 (GraphPad). Statistical analysis was conducted using Student's t-test. A statistically significant difference was considered when *p* < 0.05. All *in vitro* experiments were repeated three times, and six rats in each group for *in vivo* experiments.

## 3 Results

### 3.1 Characterization of the activity of RLMs, HLMs, and rCYP3A4 in tofacitinib treatment and screening of inhibitors

**FIGURE 1:**
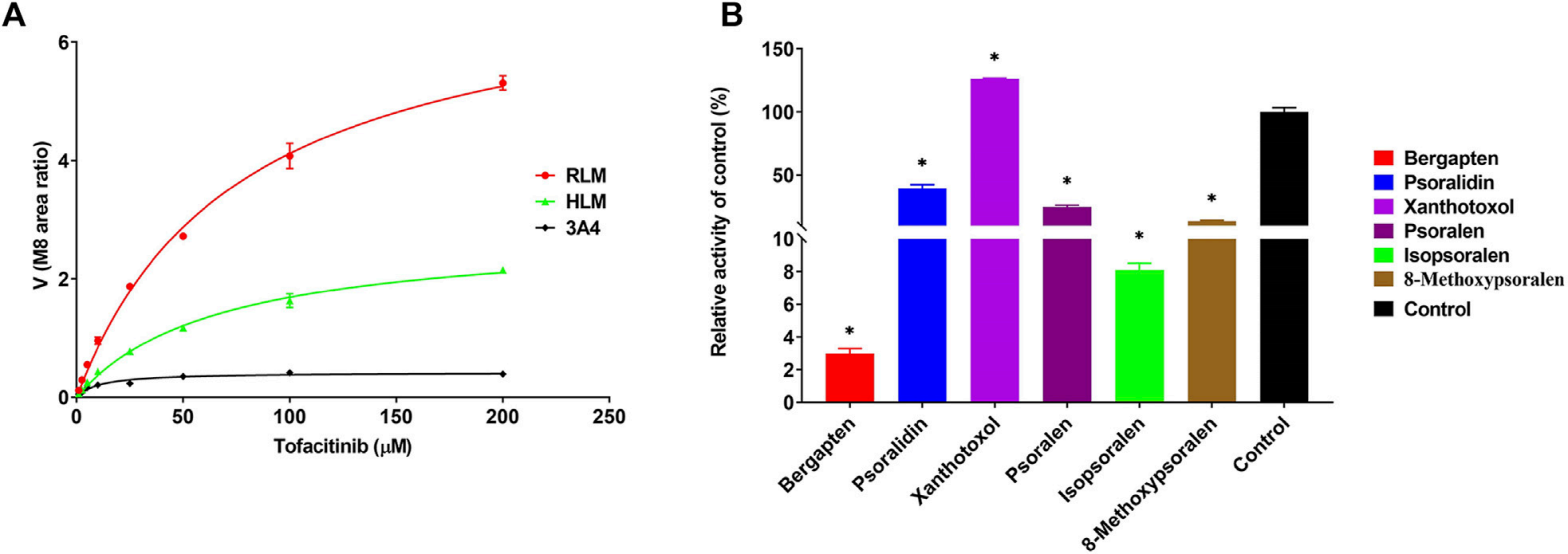
**(A)** Michaelis-Menten plots of enzymatic activity of RLMs, HLMs and rCYP3A4 on tofacitinib metabolism. Data are presented as the mean ± standard deviation of three experiments. **(B)** Screening of bergapten, psoralidin, xanthotoxol, psoralen, isopsoralen, and 8-methoxypsoralen in RLMs. Values are mean ± SD, *n* = 3. *p < 0.05, significant difference from control group.

The Michaelis-Menten curves and Michaelis kinetic parameters of tofacitinib in RLMs, HLMs and rCYP3A4 were shown in Figure 1A; Table 1. There is no significant difference between RLMs, HLMs, and rCYP3A4 based on changes in maximum velocity of reaction (*Vmax*). Our data indicated that the *Km* of tofacitinib in RLMs, HLMs and rCYP3A4 were 76.25 ± 4.514 μM, 65.85 ± 4.749 μM and 1 0.61 ± 1.667 μM. Figure 1B demonstrated the inhibitory effectiveness of six furanocoumarins on RLMs. Among them, bergapten and isopsoralen were selected for their potent inhibitory activity. And the results were statistically significant compared to the control group (*p* < 0.05).

**TABLE 1:** Kinetic parameters for the activity of RLMs, HLMs, and rCYP3A4 variants on tofacitinib metabolism.

Enzyme	*Km* (μM)	*Vmax* (M8 area ratio)
RLM	76.25 ± 4.514	7.259 ± 0.1879
HLMs	65.85 ± 4.749	2.802 ± 0.0842
rCYP3A4	10.61 ± 1.667	0.421 ± 0.0169

Notes: *n* = 3 per group; data are expressed as mean ± SD.

### 3.2 Bergapten potently inhibits the metabolism of tofacitinib in RLMs, HLMs, and rCYP3A4 with the competitive mechanism

**FIGURE 2:**
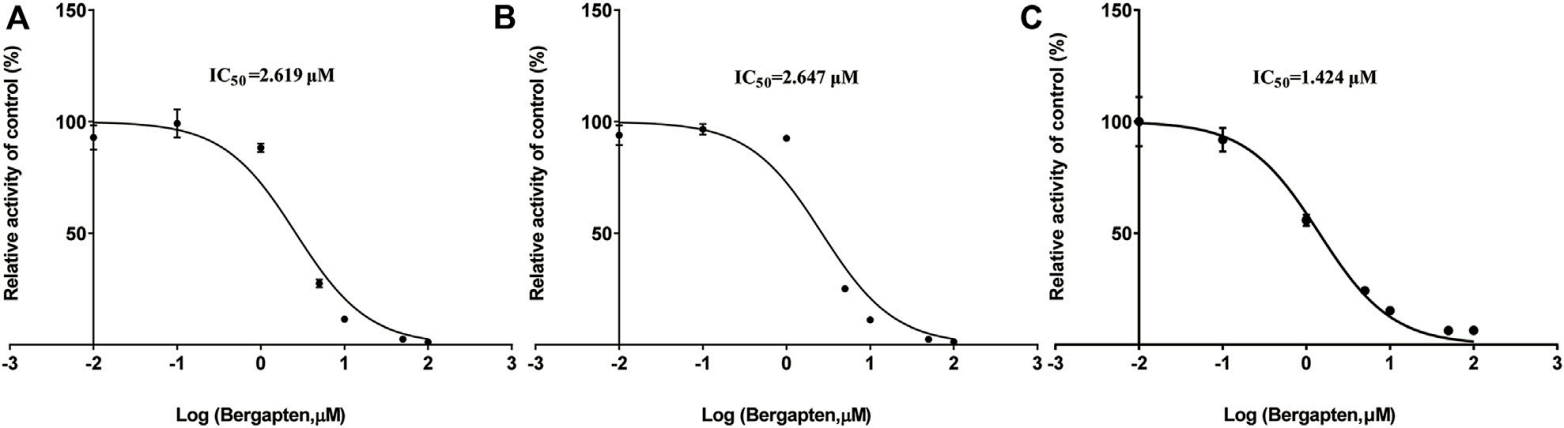
**(A)** IC_50_ of the inhibition of tofacitinib in RLMs by bergapten; **(B)** IC_50_ of the inhibition of tofacitinib in HLMs by bergapten; **(C)** IC_50_ of the inhibition of tofacitinib in rCYP3A4 by bergapten. Values are mean ± SD, *n* = 3.

**FIGURE 3:**
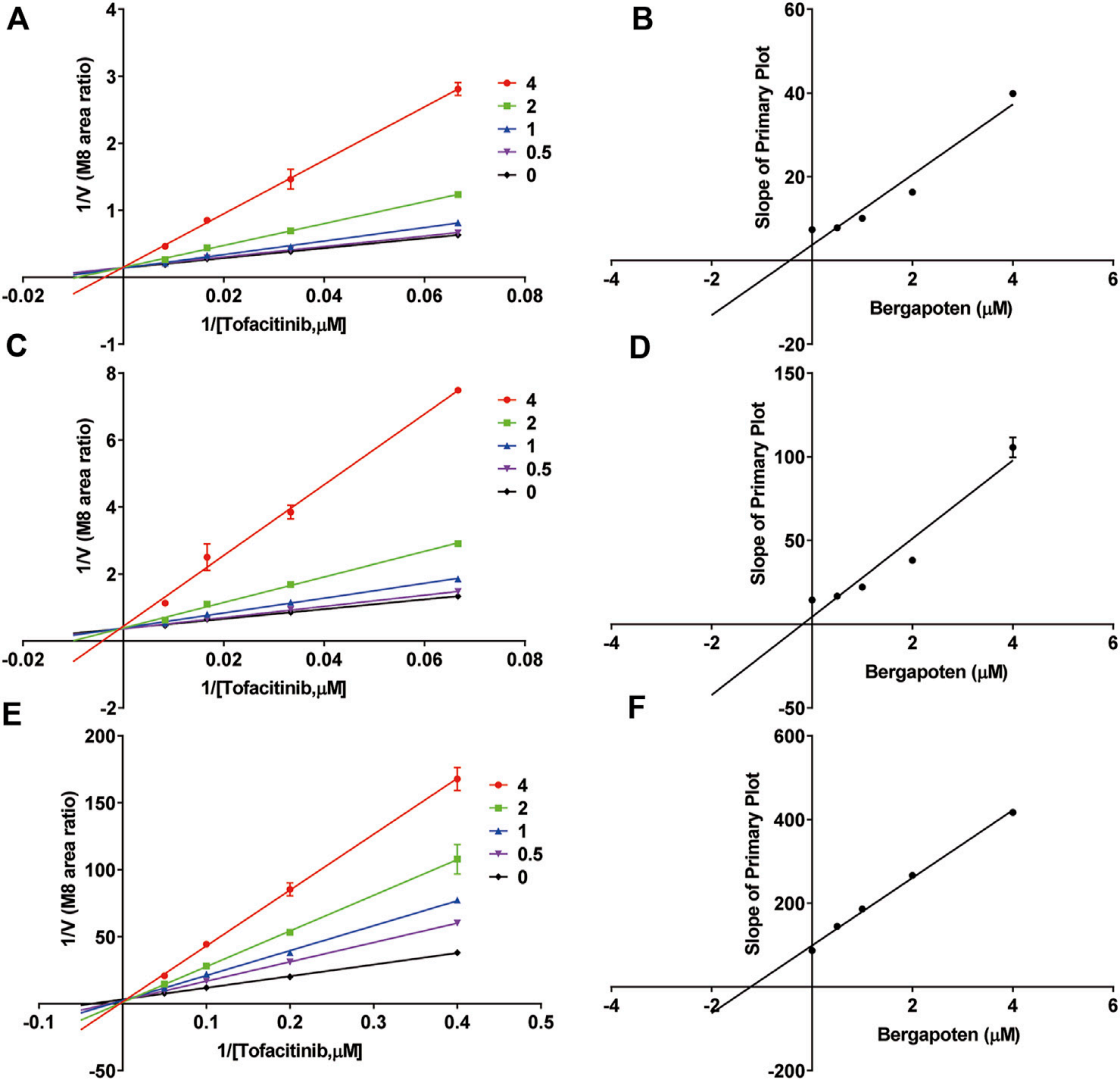
Inhibitory mechanism of bergapten against tofacitinib in **(A,B)** RLMs, **(C,D)** HLMs and **(E,F)** rCYP3A4. Values are mean ± SD, *n* = 3.

Figure 2 shows IC_50_ curves and a Lineweaver-Burk diagram of tofacitinib inhibited by bergapten in RLMs, HLMs and rCYP3A4. As given in Table 2, the results displayed that tofacitinib was restrained by bergapten in RLMs, HLMs and rCYP3A4 with IC_50_ values of 2.619 μM and 2.647 μM, 1.424 μM, respectively. All *Km* and *Vmax* values of bergapten on tofacitinib metabolism in RLMs, HLMs and rCYP3A4 are shown in Table 3. The Ki values of bergapten in RLMs, HLMs and rCYP3A4 were 0.4429 μM, 0.1893 μM, and 1.223 μM, respectively. The dynamic parameters of competitive inhibition are characterized by the increase of *Km* value and no change of *Vmax*. The findings revealed that bergapten competitively inhibited the metabolism of tofacitinib in RLMs, HLMs, and rCYP3A4 (Figure 3).

**TABLE 2:** The IC_50_ values and inhibitory effects of bergapten and isopsoralen on tofacitinib metabolism in RLMs, HLMs, and rCYP3A4.

Drug	Enzyme	IC_50_ (μM)	Inhibition type	Ki (μM)
Bergapten	RLMs	2.619	Competitive inhibition	0.4429
	HLMs	2.647	Competitive inhibition	0.1893
	rCYP3A4	1.424	Competitive inhibition	1.223
Isopsoralen	RLMs	19.27	Competitive inhibition	2.186
	HLMs	12.34	Competitive inhibition	1.774
	rCYP3A4	17.14	Competitive inhibition	15.36

**TABLE 3:** The *Km* and *Vmax* values of bergapten on tofacitinib metabolism in RLMs, HLMs, and rCYP3A4.

Enzyme	Bergapten (μM)	*Km* (μM)	*Vmax* (M8 area ratio)
RLMs	0	65.46	8.021
	0.5	72.26	8.054
	1	88.57	8.111
	2	159.6	8.689
	4	410.8	9.555
HLMs	0	47.84	2.973
	0.5	53.15	2.960
	1	76.71	3.018
	2	190.5	4.088
	4	∼5,861	∼43.85
rCYP3A4	0	25.13	0.2966
	0.5	57.43	0.4034
	1	97.19	0.5032
	2	196.9	0.7462
	4	∼3.026E+015	∼7.225E+12

### 3.3 Ispsoralen potently blocks tofacitinib metabolism in RLMs, HLMs, and rCYP3A4 with the competitive mechanism

**FIGURE 4:**
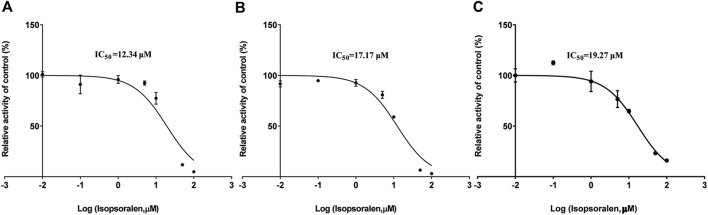
**(A)** IC_50_ of tofacitinib inhibition in RLMs by isopsoralen; **(B)** IC_50_ of tofacitinib inhibition in HLMs by isopsoralen; **(C)** IC_50_ of tofacitinib inhibition in rCYP3A4 by isopsoralen.

**FIGURE 5:**
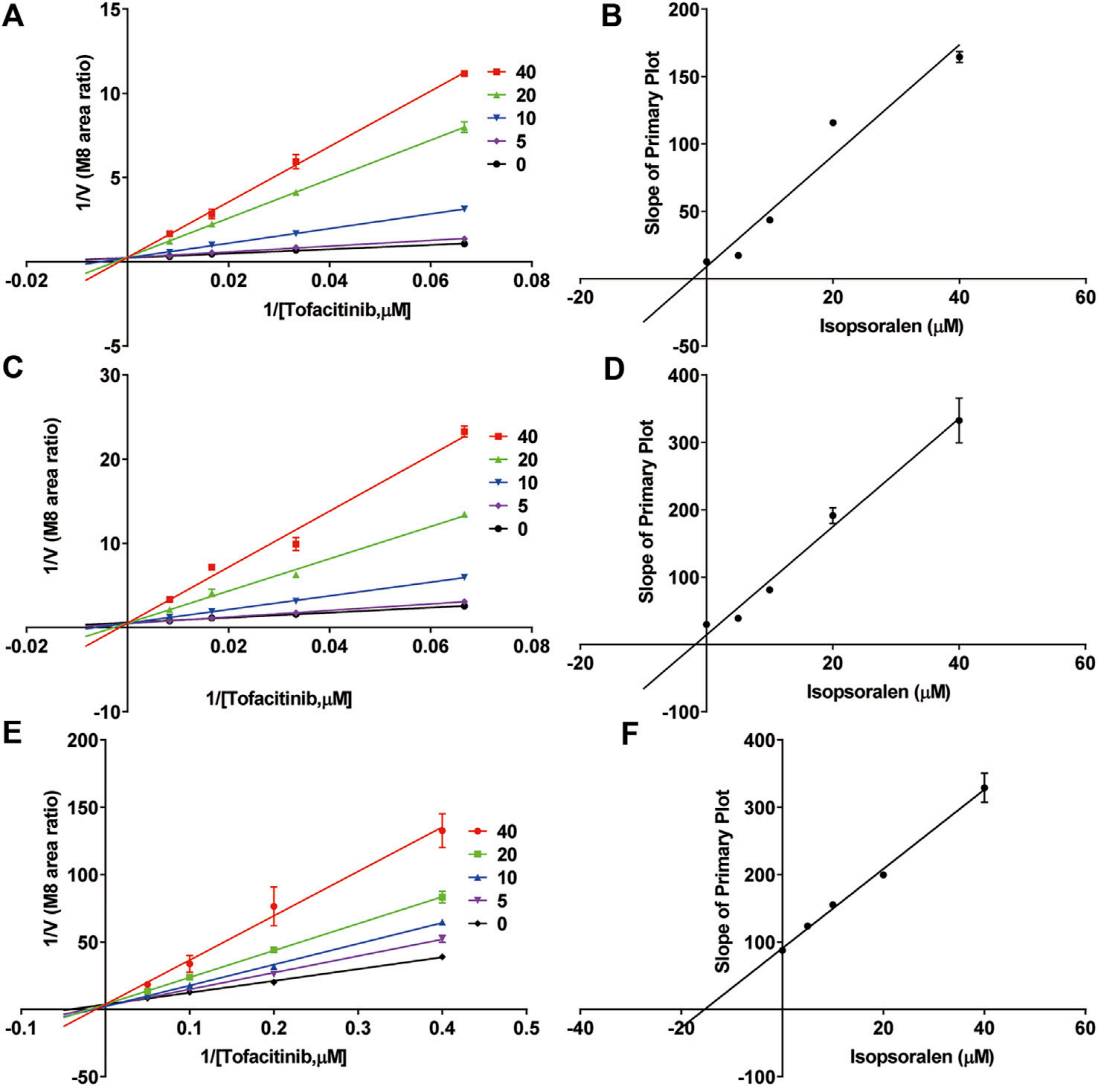
Inhibitory mechanism of isopsoralen against tofacitinib in **(A,B)** RLMs, **(C,D)** HLMs and **(E,F)** rCYP3A4. Values are mean ± SD, *n* = 3.

Figure 4; Table 2 exhibited the IC_50_ curves, Lineweaver-Burk plot and the IC_50_ and Ki values of tofacitinib metabolism by isopsoralen in RLMs, HLMs and rCYP3A4. The IC_50_ values of Isopsoralen were 12.34 μM and 17.17 μM, 19.27 μM. Table 4 shows *Km* and *Vmax* values of ispsoralen on tofacitinib metabolism in RLMs, HLMs and rCYP3A4. Isopsoralen exhibited competitive suppression of tofacitinib metabolism in RLMs, HLMs and rCYP3A4, with Ki values of 15.36 μM, 1.744 μM, and 2.186 μM (Figure 5).

**TABLE 4:** The *Km* and *Vmax* values of isopsoralen on tofacitinib metabolism in RLMs, HLMs, and rCYP3A4.

Enzyme	Isopsoralen (μM)	*Km* (μM)	*Vmax* (M8 area ratio)
RLMs	0	77.71	5.190
	5	114.5	5.555
	10	259.0	5.529
	20	537.5	4.503
	40	409.6	2.664
HLMs	0	61.72	1.886
	5	71.14	1.938
	10	134.4	1.718
	20	431.5	2.148
	40	930.4	2.589
rCYP3A4	0	20.63	0.2455
	5	38.97	0.3273
	10	40.64	0.2849
	20	68.67	0.3328
	40	142.9	0.4429

### 3.4 Molecular docking analysis of bergapten and isopsoralen on CYP3A4

**FIGURE 6:**
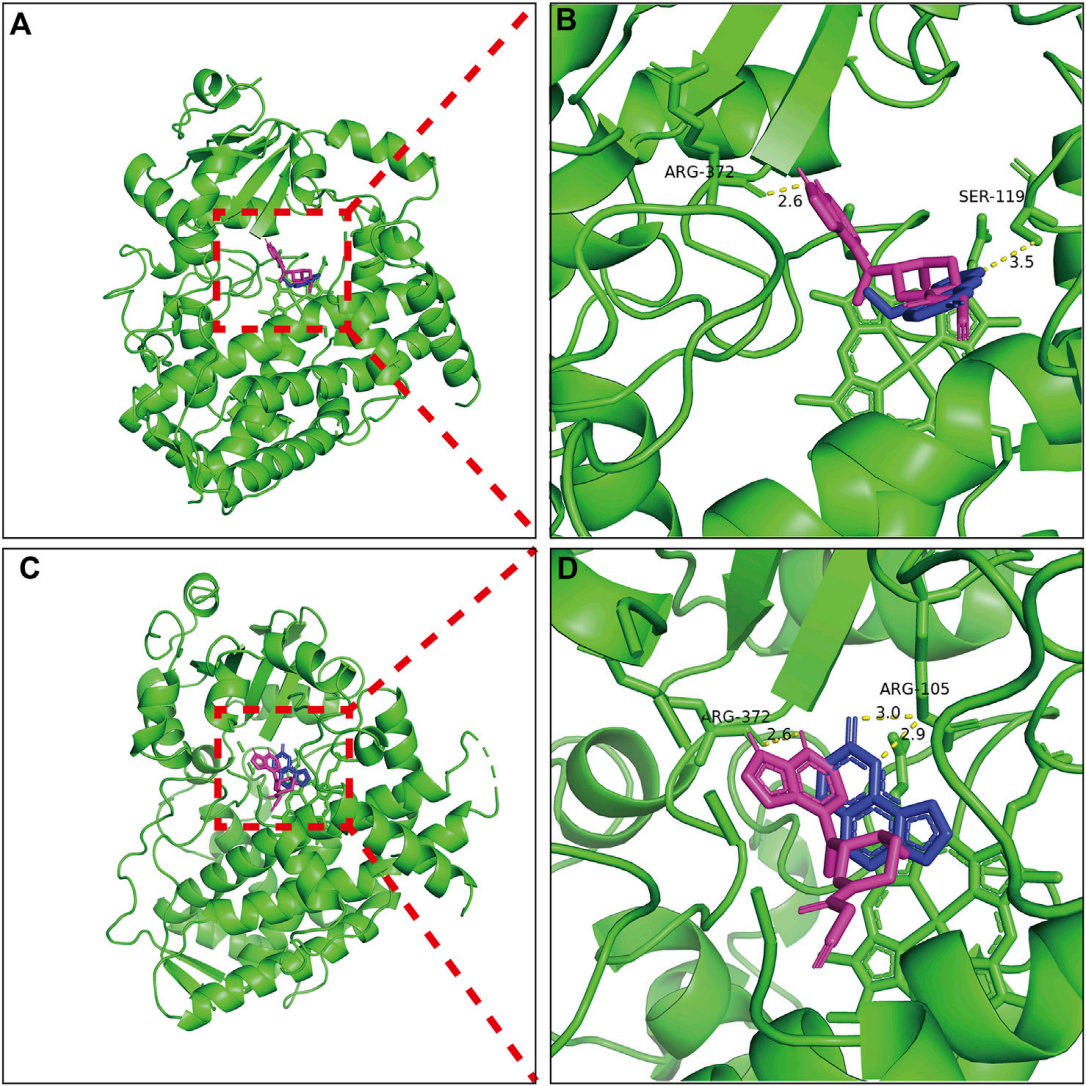
Molecular docking scheme of tofacitub and bergapten **(A)** and isopsoralen **(C)**; Action site between bergapten **(B)**, isopsoralen **(D)** and tofacitinib and CYP3A4 via hydrogen bonding.

The results of molecular docking and binding energy calculations are presented in Figure 6. Bergapten, isopsoralen and tofacitinib were well-docked at the active sites of CYP3A4. Molecular docking analysis has shown that tofacitinib, bergapten and isopsoralen establish hydrogen bond interactions with amino acid residues on CYP3A4 through hydrogen bonding. The CYP3A4 binding energies of tofacitinib, bergapten and isopsoralen were −6.8, −6.2, and −6.7 kcal/mol.

### 3.5 Effects of BGZs on the pharmacokinetics of tofacitinib *in vivo*

**FIGURE 7:**
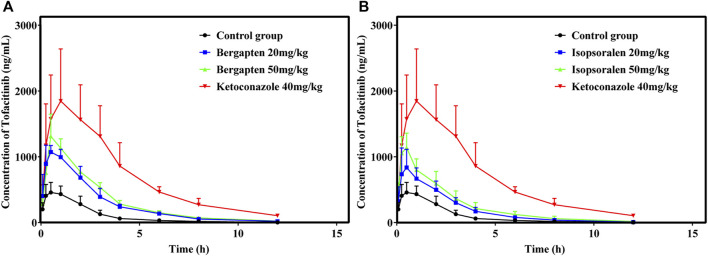
Mean plasma concentration–time curves of tofacitinib in different treatment groups. Group **(A)**, bergapten; group **(B)**, isopsoralen (*n* = 6).

Figures 7A, B showed the mean plasma concentration-time curve of the bergapten or isopsoralen treatment group or control group after oral supplementation of tofacitinib. The pharmacokinetic properties of tofacitinib were evaluated using a non-compartmental model, as presented in Tables 5, 6. We observed that tofacitinib was rapidly absorbed by the plasma and reached its maximum concentration of 509.99 mg/L at 0.5 h following oral administration. Compared with the control group, the AUC _(0-t)_, AUC _(0-∞)_, MRT _(0-t)_, MRT _(0-∞)_ and Cmax of tofatinib increased in varying degrees (all *p* < 0.05), but CLz/F decreased in varying degrees (*p* < 0.05) in the bergapten group and isopsoralen group in a dose-dependent manner. Intriguingly, there was no statistically significant difference in changes in t_1/2z_ and T_max_ (*p* > 0.05). Collectively, these findings indicated that bergapten and isopsoralen inhibited the metabolism of tofacitinib in a dose-dependent manner.

**TABLE 5:** Main pharmacokinetic parameters of tofacitinib in bergapten group and control group in rats.

Parameters	Control group	Bergapten group		Ketoconazole group
		20 mg/kg	50 mg/kg	
AUC_(0-t) (_μg/h·L)	1,228.50 ± 341.32	3,291.52 ± 545.46*	3,811.87 ± 353.38*	8,384.65 ± 2,729.10*
AUC_(0-∞)_ (μg/h·L)	1,236.65 ± 342.22	3,357.53 ± 559.47*	3,877.63 ± 364.02*	8,917.99 ± 2,795.66*
MRT_(0-t)_ (h)	2.10 ± 0.27	2.55 ± 0.14*	2.62 ± 0.16*	3.46 ± 0.30*
MRT_(0-∞) (_h)	2.19 ± 0.28	2.80 ± 0.28*	2.84 ± 0.20*	4.27 ± 0.73*
t_1/2_z(h)	1.73 ± 0.44	2.16 ± 0.37	2.13 ± 0.47	2.99 ± 0.69*
T_max_(h)	0.50 ± 0.27	0.54 ± 0.25	0.58 ± 0.20	1.17 ± 0.41*
CLz/F (L/h/kg)	8.62 ± 2.36	3.05 ± 0.49*	2.60 ± 0.27*	1.22 ± 0.39*
C_max_ (μg/L)	509.99 ± 125.41	1,108.61 ± 134.54*	1,377.20 ± 261.72*	1,891.49 ± 722.43*

Notes: *n* = 6 per group; data are expressed as mean ± SD; *p < 0.05 indicates significant differences from the control group.

**TABLE 6:** Main pharmacokinetic parameters of tofacitinib in isopsoralen group and control group in rats.

Parameters	Control group	Isopsoralen group		Ketoconazole group
		20 mg/kg	50 mg/kg	
AUC_(0-t) (_μg/h·L)	1,228.50 ± 341.32	2,346.65 ± 628.56*	3,034.50 ± 704.47*	8,384.65 ± 2,729.10*
AUC_(0-∞)_ (μg/h·L)	1,236.65 ± 342.22	2,367.48 ± 629.72*	3,080.24 ± 724.13*	8,917.99 ± 2,795.66*
MRT_(0-t)_ (h)	2.10 ± 0.27	2.40 ± 0.19*	2.51 ± 0.34*	3.46 ± 0.30*
MRT_(0-∞) (_h)	2.19 ± 0.28	2.51 ± 0.24	2.69 ± 0.42*	4.27 ± 0.73*
t_1/2_z(h)	1.73 ± 0.44	1.78 ± 0.32	1.97 ± 0.22	2.99 ± 0.69*
T_max_(h)	0.50 ± 0.27	0.54 ± 0.25	0.33 ± 0.13	1.17 ± 0.41*
CLz/F (L/h/kg)	8.62 ± 2.36	4.49 ± 1.21*	3.42 ± 0.87*	1.22 ± 0.39*
C_max_ (μg/L)	509.99 ± 125.41	882.31 ± 273.94*	1,240.38 ± 144.81*	1,891.49 ± 722.43*

Notes: *n* = 6 per group; data are expressed as mean ± SD; *p < 0.05 indicates significant differences from the control group.

## 4 Discussion

Tofacitinib is mainly metabolized by cytochrome P450 (CYP) 3A4 in the human body, followed by CYP2C19 metabolism (Veeravalli et al., 2020). Approximately 70% of tofacitinib is eliminated by a non-renal mechanism, whereas the remaining 30% is eliminated through the renal system (Bannwarth et al., 2013; Abdulrahim et al., 2019). Researchers found that the drug bergapten in *P. corylifolia* strongly blocks the activity of the CYP3A4 enzyme (Ho et al., 2001). Previous studies have found that isopsoralen can disrupt the activity of human recombinant CYP3A4 enzyme *in vitro* (Liu and Flynn, 2015; Hai et al., 2017). In this study, we measured the content of tofacitinib and its metabolite M8 to investigate the interaction of bergapten and isopsoralen with tofacitinib in rat liver microsomes, human liver microsomes and rCYP3A4. The results exhibited that bergapten and isopsoralen inhibited the metabolism of tofacitinib in rCYP 3A4, RLMs and HLMs. The relationship between substrates and inhibitors can be divided into three categories: competitive inhibition, non competitive inhibition, and uncompetitive inhibition. The dynamic parameters of competitive inhibition are characterized by the increase of *Km* value and no change of *Vmax*. Tofacitinib was shown to inhibit rCYP3A4, RLMs and HLMs competitively by bergapten and isopsoralen based on *Km*, *Vmax* and the Lineweaver-Burk plotting of double reciprocal plotting of the Mi equation. According to the literature, the risk of DDI is highest at ki value <1 µM, low at >50 µM and medium at 1–50 µM (Bjornsson et al., 2003a).Therefor, the risk of DDI between bergapten and tofacitinib was medium in 3A4 and high risk in RLMs and HLMs. In CYP3A4, RLMs and HLMs, the risk of DDI of isopsoralen and tofacitinib was medium.

The oral intervention of tofacitinib (10–100 mg/kg) to male Sprague-Dawley rats showed dose-dependent AUC above 50 mg/kg. Therefore, we selected 10 mg as the oral dose of tofacitinib (Lee and Kim, 2019). The ratio of AUCI/AUC greater than 2 indicates a high risk of DDI caused by drug interactions (Tucker et al., 2001; Bjornsson et al., 2003b). The AUCI/AUC ratios for bergapten at 20 mg/kg and 50 mg/kg were 2.7 and 3.1, respectively. Similarly, the AUCI/AUC ratios for isopsoralen were 1.91 and 2.49. Isopsoralen and bergapten increase tofacitinib-induced DDI risk. The AUC_(0-∞)_ (*p* < 0.05), Cmax (*p* < 0.05) and MRT_(0-∞)_ of tofacitinib were augmented and CLz/F (*p* < 0.05) was decreased in a dose-dependent manner after the oral intervention of bergapten and isopsoralen *in vivo*. Taken together, the results suggested that bergapten and isopsoralen might inhibit the metabolism of tofacitinib through CYP3A4 *in vivo*. And the risk of adverse reactions when bergapten or isopsoralen are combined with tofacitinib may increase due to an increase in the dosage of bergapten and isopsoralen.

Hydrogen bonding stabilizes energetically favorable ligands in protein structures. The interaction energy between target 3D structures and ligand molecules must be quantified to analyze the affinity of the binding (Patil et al., 2010). At present, it is generally believed that when the binding energy value is less than −5 kJ/mol, the binding between ligand and receptor is relatively stable, and the lower the binding energy values, the lower the conformational stability energy, and the greater the possibility of interaction between receptor and ligand. According to the formula 1 kJ = 0.24 kcal, the unit conversion between kcal/mol and kJ/mol shows that the binding energy of bergapten, isopsoralen, tofacitinib and CYP3A4 is less than −5 kJ/mol. The binding energy of bergapten, isopsoralen and tofacitinib is almost the same, which indicates that in the interaction with CYP3A4, bergapten and isopsoralen have similar stable binding ability to CYP3A4 as tofacitinib. In addition, according to the molecular docking results, it can be seen that the binding sites of bergapten and isopsoralen with CYP3A4 are similar to those of tofacitinib. This confirms the results of *in vitro* and *in vivo* studies that bergapten and isopsoralen may competitively inhibit tofacitinib metabolism in CYP3A4.

The dosage and metabolism of DDIs might produce adverse drug reactions. It is widely believed that drug metabolism plays an important role in drug safety. Therefore, it is necessary to evaluate the risk of adverse reactions between drugs due to dosage and metabolism. This study confirmed the inhibitory effect of bergapten and isopsoralen on the metabolism of tofacitinib *in vitro* and *in vivo*. The CYP450 enzyme inhibitors burgapten and isopsoralen increase blood tofacitinib levels *in vivo. P. corylifolia* possesses significant application potential owing to its abundant pharmacological action. This indicates that when we are using tofacitinib and *P. corylifolia* for combined treatment of rheumatoid arthritis, attention should be paid to the dosage of *P. corylifolia* intake.

Tofacitinib and bergapten/isopsoralen have significant interactions, which can be used to guide clinical medication and treatment in patients with RA. While further studies are required due to rat-human species differences.

## 5 Conclusion

DDIs may result in adverse drug reactions depending on dosage and metabolism. Drug metabolism is a crucial factor in maintaining drug toxicity. This study confirmed the inhibitory effect of bergapten and isopsoralen on CYP3A4 *in vitro* and vivo. We found that bergapten and isopsoralen exhibited distinct regulatory impacts on the metabolism of tofacitinib in RLM, HLM and CYP3A4. Our results also revealed that berkatten and isopsoralen inhibited CYP450 enzymes to enhance blood tofacitinib levels *in vivo*. Tofacitinib exposure may lead to more severe and frequent adverse events. Therefore, when using tofacitinib to treat rheumatoid arthritis, it is necessary to pay attention to or adjust the dose of Chinese herbs containing bergapten and isopsoralen, so as to reduce the occurrence of adverse reactions caused by the increase of tofacitinib blood concentration. This study provides a theoretic basis for the combination of *P. corylifolia* and tofacitinib. In addition to *P. corylifolia*, there are also many other traditional Chinese medicines that contain bergapten and isopsoralen, such as Coastal Glehnia Root and Saposhnikoviae Radiox.This study also provides significance for combining use of tofacitinib and traditional Chinese medicine containing this type of coumarin in the treatment of diseases.

## Data Availability

The original contributions presented in the study are included in the article/Supplementary material, further inquiries can be directed to the corresponding authors.
